# Comprehensive Clinicopathologic, Immunohistochemical, and Genomic Profiling of Sporadic Ampullary Somatostatin-producing D-cell Neuroendocrine Tumors Identifies Recurrent *HRAS* Hotspot Mutations

**DOI:** 10.1007/s12022-026-09934-y

**Published:** 2026-09-12

**Authors:** Alessandro Vanoli, Erica Travaglino, Frediano Inzani, Tommaso Orione, Federica Grillo, Paola Parente, Paola Spaggiari, Silvia Uccella, Alessandro Zerbi, Anna Caterina Milanetto, Mauro Lecca, Andrea Peri, Antonio Di Sabatino, Marco Paulli, Stefano La Rosa, Matteo Fassan, Edoardo Errichiello

**Affiliations:** 1 Department of Molecular Medicine, Unit of Anatomic Pathology, University of Pavia, Pavia, Italy; 2 Anatomic Pathology Unit, Fondazione IRCCS Policlinico San Matteo, Pavia, Italy; 3 Department of Surgical and Integrated Diagnostic Sciences, University of Genova, Genoa, Italy; 4 Anatomic Pathology Unit, AOM IRCCS Ospedale Policlinico San Martino, Genoa, Italy; 5 Pathology Unit, Azienda Ospedaliero-Universitaria Policlinico Riuniti Foggia, Foggia, Italy; 6 Pathology Unit, IRCCS Humanitas Research Hospital, Rozzano, Milan Italy; 7 Department of Biomedical Sciences, Humanitas University, Pieve Emanuele, Milan, Italy; 8 Pancreatic Surgery Unit, IRCCS Humanitas Research Hospital, Milan, Italy; 9 UniCamillus International Medical University in Rome, Rome, Italy; 10 Pancreatic and Digestive Endocrine Surgery, Department of Surgery, Oncology and Gastroenterology, University of Padua, Padua, Italy; 11 Department of Molecular Medicine, Unit of Medical Genetics, University of Pavia, Pavia, Italy; 12 Department of Surgery, University of Pavia, Pavia, Italy; 13 Surgery 2 Unit, Fondazione IRCCS Policlinico San Matteo, Pavia, Italy; 14 Department of Internal Medicine and Medical Therapeutics, University of Pavia, Pavia, Italy; 15 Department of Internal Medicine, San Matteo Hospital Foundation, Pavia, Italy; 16 Unit of Pathology, Department of Medicine and Technological Innovation, University of Insubria, Varese, Italy; 17 Hereditary Cancer Research Center, Department of Medicine and Technological Innovation, University of Insubria, Varese, Italy; 18 Unit of Pathology, ASST Dei Sette Laghi, Varese, Italy; 19 Veneto Institute of Oncology (IOV-IRCCS), Padua, Italy; 20 Department of Medicine (DIMED), Surgical Pathology Unit, University of Padua, Padua, Italy; 21 Neurogenetics Research Center, IRCCS Mondino Foundation, Pavia, Italy

**Keywords:** Ampulla of Vater, Amphicrine tumors, Major papilla, Neuroendocrine neoplasms, Targeted therapy

## Abstract

Ampullary somatostatin-producing D-cell neuroendocrine tumors are rare neoplasms that may be associated with type 1 neurofibromatosis. The molecular features of sporadic ampullary somatostatin-producing D-cell neuroendocrine tumors (SAMSOM-NETs) remain poorly characterized. We performed an integrated morphological, immunohistochemical, and genomic analysis of a multicenter series of SAMSOM-NETs. Eleven cases were included (73% male; median age: 63 years). All six patients who underwent lymphadenectomy were staged as pN1, and liver metastases were found in three cases; however, no tumor-related deaths occurred (median follow-up: 104 months). Common histologic features that can pose diagnostic challenges in the differential diagnosis with adenocarcinoma included a tubulo-glandular architecture (100%), periodic Acid–Schiff (PAS)-positive intraluminal mucin (73%), MUC1 expression (100%), and carcinoembryonic antigen (45%) expression. All tumors exhibited dot-like cytoplasmic reactivity for cytokeratins (CK) CAM5.2 or CK AE1/AE3, and 82% were CK7-positive. ISL1 and PDX1 were diffusely expressed in all cases, while CDX2 was positive in 54% and ARX showed only focal expression in four tumors. Genomic profiling revealed microsatellite stability and low tumor mutational burden. Alterations in the RAS pathway, including *HRAS* mutations (4 cases, 36%), a *KRAS* mutation (1 case), and *NF1* alterations (one case), were identified in 54% of cases and in all tumors with liver metastases. Additional molecular findings included a *CDK12* splice-site alteration and an *NTRK3*::*PRDM4* fusion. Potentially actionable alterations affecting kinase-related pathways were detected in 64% of tumors. Our findings support SAMSOM-NET as a peculiar neuroendocrine tumor subtype showing distinctive histologic and molecular characteristics with potential diagnostic and therapeutic implications.

**Supplementary Information:**

The online version contains supplementary material available at https://doi.org/10.1007/s12022-026-09934-y.

## Introduction

Neuroendocrine tumors (NETs) of the ampullary region are rare neoplasms, accounting for approximately 1% of malignant tumors arising in this anatomic site [1]. These tumors, which include both NETs of the ampulla of Vater and the even rarer NETs originating from the minor papilla (accessory ampulla) [2], should be distinguished from non-ampullary duodenal NETs, both clinically and histologically. A recent systematic review and meta-analysis reported a pooled prevalence of lymph node metastasis of 46.8% in ampullary NETs, compared with 9.5% in non-ampullary duodenal NETs [3]. While non-ampullary duodenal NETs are predominantly G cell tumors, the most common histological subtype of ampullary NETs is the somatostatin-expressing D-cell neuroendocrine tumor [4, 5]. Duodenal/ampullary somatostatin-producing NETs have also long been referred to as “somatostatinomas”; however, this term is inappropriate because their association with the full somatostatinoma syndrome is exceptional [4, 6].

Ampullary somatostatin-producing NETs differ from both non-ampullary duodenal somatostatin-producing NETs and other ampullary NETs. Compared to non-ampullary duodenal somatostatin-producing NETs, ampullary somatostatin-producing NETs exhibit more aggressive histological features and greater propensity for lymph node metastasis compared with their non-ampullary duodenal counterparts [5]. Moreover, while somatostatin-producing NETs associated with multiple endocrine neoplasia type 1 (MEN1) typically arise in the non-ampullary duodenum, those associated with neurofibromatosis type 1 (NF1) and Pacak–Zhuang syndrome have been reported more frequently in the ampullary region [4, 7]. Compared to remaining ampullary NETs, ampullary somatostatin-producing NETs are diagnosed at a younger age and are more frequently associated with NF1; they also tend to present as larger tumors [8]. In addition to somatostatin expression, ampullary somatostatin-producing NETs display distinctive morphological and immunophenotypic features, which include the almost constant presence of a tubuloacinar/pseudoglandular pattern, often with intraluminal psammoma bodies, and the frequent expression of MUC1 [9].

Collectively, these features support ampullary somatostatin-producing NET as a distinctive tumor subtype warranting integrated histologic and molecular investigation. Although one study has molecularly characterized a subset of NF1-associated duodenal/ampullary NETs, highlighting the role of chromosome 22 deletion or loss of heterozygosity and somatic inactivation of the wild-type *NF1* allele in tumor development [10], and “ampullary somatostatinomas” associated with Pacak–Zhuang syndrome have been shown to harbor somatic gain of function *EPAS1/HIF2A* variants [11], the genomic landscape of sporadic ampullary somatostatin-producing D-cell NETs (SAMSOM-NETs) remains unknown.

The aim of our study was to conduct an integrated morphological, immunohistochemical, and genomic analysis of a multicenter series of surgically resected SAMSOM-NETs. Specifically, we aimed to: (i) further explore the morphological features of these tumors and characterize their immunophenotypic profile, with particular emphasis on the potential amphicrine/amphicrine-like characteristics of the tumor cells, and (ii) explore the genomic alterations occurring in these unique NETs. This comprehensive approach aims to deepen our understanding of the key characteristics of SAMSOM-NETs.

## Materials and Methods

### Study Population and Clinico-pathological Data

Cases were collected from endocrine/neuroendocrine tumor registries of the Departments of Pathology of IRCCS San Matteo Hospital (Pavia), IRCCS Humanitas Research Hospital (Milan), University Hospital of Padua, and AOM San Martino Hospital (Genoa). Only cases fulfilling the following criteria were included: (i) a primary NET arising from the major or minor duodenal ampulla; (ii) diffuse somatostatin immunoreactivity, defined as somatostatin expression in > 50% of neoplastic cells, according to the criteria adopted by the World Health Organization (WHO) 6th edition classification of digestive system tumors for somatostatin cell duodenal and ampullary NETs [12]; (iii) availability of formalin-fixed paraffin-embedded (FFPE) tumor tissue block; and (iv) absence of clinical or genetic evidence of hereditary cancer syndromes. A total of 11 SAMSOM-NETs diagnosed between 2007 and 2022 were included. Clinical data (patient age at diagnosis, sex, symptoms, tumor location, clinical stage, follow-up information) were obtained from medical records, institutional tumor registries, and interviews with the treating physicians. Each case was centrally reviewed by a pathologist with expertise in gastrointestinal and endocrine pathology (A.V.). The study was conducted in accordance with the clinical standards set forth in the 1975 Declaration of Helsinki and its revisions, and was approved by the Ethics Committee of Pavia (protocol number: 20210027824).

### Histological Characterization

All available Hematoxylin and Eosin (H&E)-stained slides of each case were histologically reviewed for the following parameters (according to College of American Pathologists Protocol criteria): mitotic count (expressed as number of mitoses per 2 mm^2^); tumor size; extent of local invasion; architectural pattern; presence of psammoma bodies; stroma characteristics; lymphatic invasion; vascular invasion; perineural invasion; tumor necrosis, [13]. Tumor pathological stage was assigned according to the American Joint Committee on Cancer (AJCC) TNM classification system (9th edition) [14].

Architectural patterns were categorized as solid, trabecular, and tubulo-glandular [15, 16]. The predominant pattern was recorded for each case. Extent of tubulo-glandular structures was classified as focal (< 25% of tumor), substantial (25–75%) and diffuse (> 75%). The presence of cribriform and thyroid-like follicular structures containing inspissated, eosinophilic colloid-like material was also assessed and, if present, recorded as focal or diffuse.

### Histochemistry

Histochemical analyses included Periodic Acid–Schiff with Diastase digestion (PAS-D), mucicarmine, and Congo red stainings (Dako/Agilent Technologies, Santa Clara, CA, USA), performed on the Dako Agilent Artisan Link Pro platform. PAS-D staining was assessed in all cases for tumor cell cytoplasmic or apical positivity, intraluminal positivity, and staining distribution (focal vs diffuse), whereas mucicarmine staining was performed in selected cases exhibiting thyroid-like follicular structures to highlight acid mucins. Congo red staining was carried out in tumors showing stromal features suggestive of amyloid deposits; cases with Congo red positive stroma were subsequently evaluated under polarized light microscopy to assess for the green birefringence diagnostic of amyloid.

### Immunohistochemical Characterization

Immunohistochemical staining was performed using a fully automated system (BenchMark Ultra; Ventana Medical Systems). The following immunohistochemical tests were performed (the specifics of the utilized antibodies are summarized in Supplementary Table 1): (i) *General neuroendocrine markers*, i.e., chromogranin A, synaptophysin, INSM1, and somatostatin receptor type 2 A (SSTR2A); (*ii*) Ki-67; *(iii) cytokeratins (CK)*, i.e., CK CAM5.2, CK AE1/AE3, CK7, and CK20; *(iv) mucins* (MUC1/EMA, MUC2, MUC5AC, MUC6)*, mCEA and BCL10; (v) neural crest markers* (i.e., S100, SOX10), *sympatico-adrenal markers* (i.e., GATA3, TH, and PHOX2B) and *neurofilament protein*; *(vi) transcription factors*, i.e. ARX, CDX2, ISL1, PDX1, SATB2, TTF-1, estrogen receptor and progesterone receptor; (vii) *hormones*, i.e., somatostatin, gastrin, serotonin, insulin, glucagon, pancreatic polypeptide, and calcitonin; (viii) *other markers*, i.e. succinate dehydrogenase, subunit B (SDHB), ∝ -inhibin, β-catenin, E-cadherin, and pan-TRK.

Immunohistochemical markers were assessed using three assessment approaches, according to the type of marker and previously established criteria. (i) For chromogranin A, synaptophysin, INSM1, CKs, mucins, mCEA, BCL10, neural crest and sympatho-adrenal markers (except S100), neurofilament protein, transcription factors, hormones, pan-TRK, α-inhibin, staining was first assessed as positive or negative. Positive cases were further characterized according to staining distribution (focal vs. diffuse) and intensity (weak vs. strong), following the approach described by Delfin et al. [17]. For CKs, the proportion of tumor cells showing paranuclear dot-like staining was also recorded. A series of 20 surgically resected pancreatic NETs (Pan-NETs) was tested for CK CAM5.2 for comparison. Fisher’s exact test was used to compare the proportion of SAMSOM-NETs and Pan-NETs showing paranuclear dot-like CK CAM5.2 positivity; *p* < 0.05 was considered statistically significant. Mucin and mCEA staining was additionally characterized according to localization (apical, intracytoplasmic, and/or intraluminal/psammoma bodies). The percentage of somatostatin-positive cells was also recorded. Pan-TRK was assessed only in the case harboring an *NTRK3* fusion. SDHB expression was assessed as retained or lost. (ii) Semiquantitative scoring systems based on predefined numerical thresholds were applied to SSTR2A, S100, β-catenin, and E-cadherin, according to previously published criteria. SSTR2A was scored according to Volante et al. [18], while S100 expression was assessed using a 0–3 + scoring system, as previously described [19]. The presence of sustentacular cells highlighted by S100 and/or SOX10 was also recorded. β-catenin and E-cadherin were scored for loss of membranous expression (0–3 +) as follows: 0, complete loss; 1 +, expression in < 20% of cells; 2 +, expression in 20–50% of cells; and 3 +, expression in > 50% of cells, as previously described [20]. For β-catenin, aberrant nuclear localization was additionally assessed as focal or diffuse. (iii) A percentage-based assessment was used for Ki-67. The Ki-67 proliferation index was reassessed on camera-captured images of areas with the highest proliferative activity (500–2000 tumor cells), and tumor grade was assigned according to the WHO 6th edition classification of digestive system tumors [12].

### Molecular Characterization

#### Next Generation Sequencing (NGS)

FFPE tumor samples were analyzed in a tumor-only setting using the QIAseq Multimodal Human Pan-Cancer Panel (QIAGEN, Hilden, Germany), a targeted DNA/RNA sequencing assay, covering 549 cancer-related genes for DNA variant analysis and 56 genes for RNA fusion detection. Genomic DNA and total RNA were extracted using the Maxwell automated extraction system (Promega, Madison, WI, USA) according to the manufacturer’s instructions. Pre-analytical quality control (QC) of extracted nucleic acids was performed using fluorometric quantification (Qubit 2.0 fluorometer, Thermo Fisher Scientific, Waltham, MA, USA) and capillary electrophoresis (TapeStation system 2200, Agilent Technologies, Santa Clara, CA, USA) prior to library preparation. Library preparation was performed following standard protocols, and sequencing was carried out on an Illumina NextSeq 1000/2000 platform (Illumina Inc., San Diego, CA, USA).

The assay enabled the detection of single nucleotide variants (SNVs), insertions and deletions (indels), gene fusion events, copy number variations (CNVs), as well as the estimation of tumor mutational burden (TMB) and microsatellite instability (MSI).

SNVs and indels were identified using the QIAGEN GeneGlobe data analysis workflow, including UMI-based error correction, read alignment, and variant calling. Only variants with a variant allele frequency (VAF) ≥ 2% and sufficient sequencing depth were retained for downstream analysis. Gene fusion detection was performed using a dedicated RNA fusion-calling algorithm within the GeneGlobe workflow, based on split-read and spanning-read evidence, and retained only when passing standard quality filters supported by adequate sequencing evidence. CNVs, TMB, and MSI were evaluated using QIAGEN CLC Genomics Workbench based on normalized read depth analysis and dedicated analytical modules. An exploratory loss of heterozygosity (LOH) assessment was performed in selected LOH-informative genes (*APC*, *BAP1*, *BRCA1*, *BRCA2*, *CDH1*, *CDKN2A*, *CDC73*, *FH*, *FLCN*, *MEN1*, *MLH1*, *MSH2*, *MSH6*, *NF1*, *NF2*, *PTEN*, *RB1*, *SDHA*, *SDHB*, *SDHC*, *SDHD*, *SMARCB1*, *STK11*, *TSC1*, *TSC2*, *TP53*, *VHL*, *WT1*) by evaluating pathogenic and likely pathogenic variants with marked allelic enrichment (VAF ≥ 60%) in the context of gene-specific allelic concordance patterns derived from variant allele frequency distributions. Variant interpretation was performed using an integrated stepwise classification approach previously described by our group [21], incorporating curated database evidence and the VarSome somatic classification framework. Variants were categorized as pathogenic/likely pathogenic or variants of uncertain significance (VUS) according to this system. Gene fusions and CNV were interpreted based on known oncogenic mechanisms and curated database annotations, with pathogenicity assigned when involving established oncogenic drivers or tumor suppressor genes. For comparisons between molecularly defined subgroups, continuous variables were analyzed using the Mann–Whitney U test, whereas categorical variables were compared using Fisher’s exact test. Given the limited sample size, all analyses were considered exploratory. A two-sided *p* value < 0.05 was considered statistically significant.

#### Sanger Sequencing

Sanger sequencing was performed to validate the somatic origin of two *NF1* variants identified by NGS. Briefly, 50 ng of genomic DNA extracted from SAMSOM-NET tumor tissue and matched non-neoplastic tissue (unaffected mucosa) was amplified by PCR using the EmeraldAmp GT PCR Master Mix (TaKaRa Bio Inc., Kusatsu, Japan). Amplification conditions consisted of 40 cycles at 98 °C for 10 s, 58 °C for 30 s, and 72 °C for 30 s. The primer sequences used were as follows: *NF1* intron 1 (5′-GTCATGATTTTCAATGGCAA-3′ and 5′-CCTTGTTGTGCTCAGTACTGA-3′) and *NF1* exon 21 (5′-CCTGCTCTGTATCCAATGCT-3′ and 5′-GGCTTATTTCAAACAAGTCACTCT-3′). Sanger sequencing was carried out on a 3500 Series Genetic Analyzer (Thermo Fisher Scientific, Waltham, MA, USA).

## Results

### Clinico-pathologic and Histological Features

Eleven patients with SAMSOM-NETs were included in this study, and their main clinico-pathologic and histological features are shown in Table 1.

**Table 1:** Clinicopatological and histologic data of the 11 sporadic ampullary somatostatin-producing D-cell neuroendocrine tumors examined

Case number	Age	Sex	Clinical presentation	Type of surgical resection	Tumor site	Ki-67	WHO grade	LI	PNI	Tumor size (mm)	Level of invasion	AJCC TNM	AJCC PSG	Predominant histologic structure	Tubulo-glandular structures	Stroma	PBs	PAS-D	Status at last follow- up (mo)
#1	59	M	Abdominal pain and pruritus	Pancreatico-duodectomy	Major ampulla	2%	1	Yes	No	28	Pancreas and PAT	pT3 pN1 cM0	III	TG	Substantial	Scant	Yes	Pos (focal, IL + AP)	Alive without disease (159)
#2	77	M	Unknown	Local resection	Major ampulla	1%	1	Yes	No	23	PAT	pT3 cN0^ pM1a	IV	TG	Substantial	Amyloid	Yes	Pos (focal, IL)	Dead of other causes (46)
#3	49	M	Gastrointestinal bleeding and anemia	Local resection	Major ampulla	3.7%	2	Yes	No	16	Duodenal submucosa	pT2 cN0^ cM0	NA	TR	Substantial	Amyloid	No	Neg	Alive without disease (75)
#4	64	F	Pancreatic exocrine insufficiency	Minor ampullectomy	Minor ampulla	1%	1	No	No	15	DMP	pT2 cN0^ cM0	NA	TG	Diffuse	Amyloid	Yes	Pos (focal, IL + AP)	Alive without disease (222)
#5	70	M	Abdominal pain	Transduodenal tumor excision	Minor ampulla	0.4%	1	Yes	No	7	Duodenal submucosa	pT2 cN0^ cM0	NA	N	Substantial	Scant	No	Neg	Alive without disease (93)
#6	52	F	Unknown	Pancreatico-duodectomy	Major ampulla	3%	2	Yes	Yes	25	Pancreas	pT3 pN1 cM0	III	TG	Diffuse	Scant	Yes	Pos (focal, IL + AP)	Alive without disease (201)
#7	58	M	Incidental finding on ultrasound	Ampullectomy	Major ampulla	2%	1	No	No	15	Oddi’s sphincter	pT2 cN0^ cM0	NA	TR	Focal	Scant	No	Neg	Alive without disease (121)
#8	57	M	Gastrointestinal bleeding	Ampullectomy + lymphadenectomy	Major ampulla	2%	1	Yes	Yes	32	DMP	pT2 pN1 cM0	III	N	Substantial	Amyloid	Yes	Pos (focal, IL + AP)	Alive without disease (35)
#9	63	M	Abdominal pain and jaundice	Pancreatico-duodectomy	Major ampulla	4%	2	Yes	No	35	DMP	pT2 pN1 cM0	III	N	Focal	Scant	No	Pos (focal, IL)	Alive without disease (135)
#10	72	M	Jaundice and anemia	Pancreatico-duodectomy	Major ampulla	1%	1	Yes	Yes	43	DMP	pT2 pN1 pM1a	IV	TG	Diffuse	Scant	Yes	Pos (focal, IL + AP)	Alive with disease (36)
#11	67	F	Abdominal pain and weight loss	Pancreatico-duodectomy*	Minor ampulla	6%	2	Yes	Yes	34	PAT	pT3 pN1 pM1a	IV	TG	Diffuse	Fibrous/Desmoplastic	Yes	Pos (focal, IL + AP)	Alive with disease (104)

AP = Apical; DMP = Duodenal Muscolaris Propria; IL = Intraluminal; LI: Lymphatic invasion; N = Nests; Mo = Months: NA = Not assigned; PAS-D = Periodic Acid-Schiff Diastase stain**;** PAT = Peripancreatic Adipose Tissue; PBs = Psammoma bodies; PNI: Perineural invasion; PSG: (pathologic) Prognostic stage group; TG = Tubuloglandular; TR = Trabecular. ^refers to the preoperative imaging evaluation in patients who did not undergo lymphadenectomy.*the patient received systemic therapy (everolimus) before pancreaticoduodenectomy

The median age of patients was 63 years (range: 49–77), and the majority were males (73%). Five patients underwent pancreaticoduodenectomy, while six patients had surgical local resection or surgical ampullectomy, with (1 case) or without (5 cases) lymphadenectomy. The tumor arose in the major ampulla in 8 cases and in the minor papilla in the remaining three cases. The most common clinical presentations were abdominal pain (*n* = 4), jaundice (*n* = 2), and gastrointestinal bleeding (*n* = 2). In one patient (case #7), the ampullary tumor was incidentally detected by ultrasound during follow-up for essential thrombocythemia. A prior cholecystectomy for cholelithiasis was reported in three patients (cases #4, #5 and #6), while one patient (case #7) underwent cholecystectomy for cholelithiasis concomitant with ampullectomy; however, none of the patients exhibited the classic triad of somatostatinoma syndrome or met its diagnostic criteria [12]. Preoperative plasma somatostatin levels were not available. None of the patients had a documented personal or family history of paragangliomas or polycythemia.

Seven SAMSOM-NETs were G1 while four were G2 (Ki-67 index ranging from 3 to 6%); no tumor showed a Ki-67 ≥ 10%. Tumor necrosis was absent. Lymphatic invasion and perineural invasion were found in 9 (82%) and 4 (36%) cases, respectively, whereas venous vascular invasion was not identified in any case.

Median tumor size was 2.5 cm (range 0.7–4.3). Seven (64%) tumors were staged as pT2 while four (36%) as pT3. All six cases with lymphadenectomy were categorized as pN1. Three patients had synchronous, biopsy-proven liver metastases (pM1a) with histologic features similar to the primary tumors; none of the liver metastases were surgically resected. Most cases (64%) were classified as AJCC v.9 pathologic prognostic stage group III or IV.

**Fig. 1:**
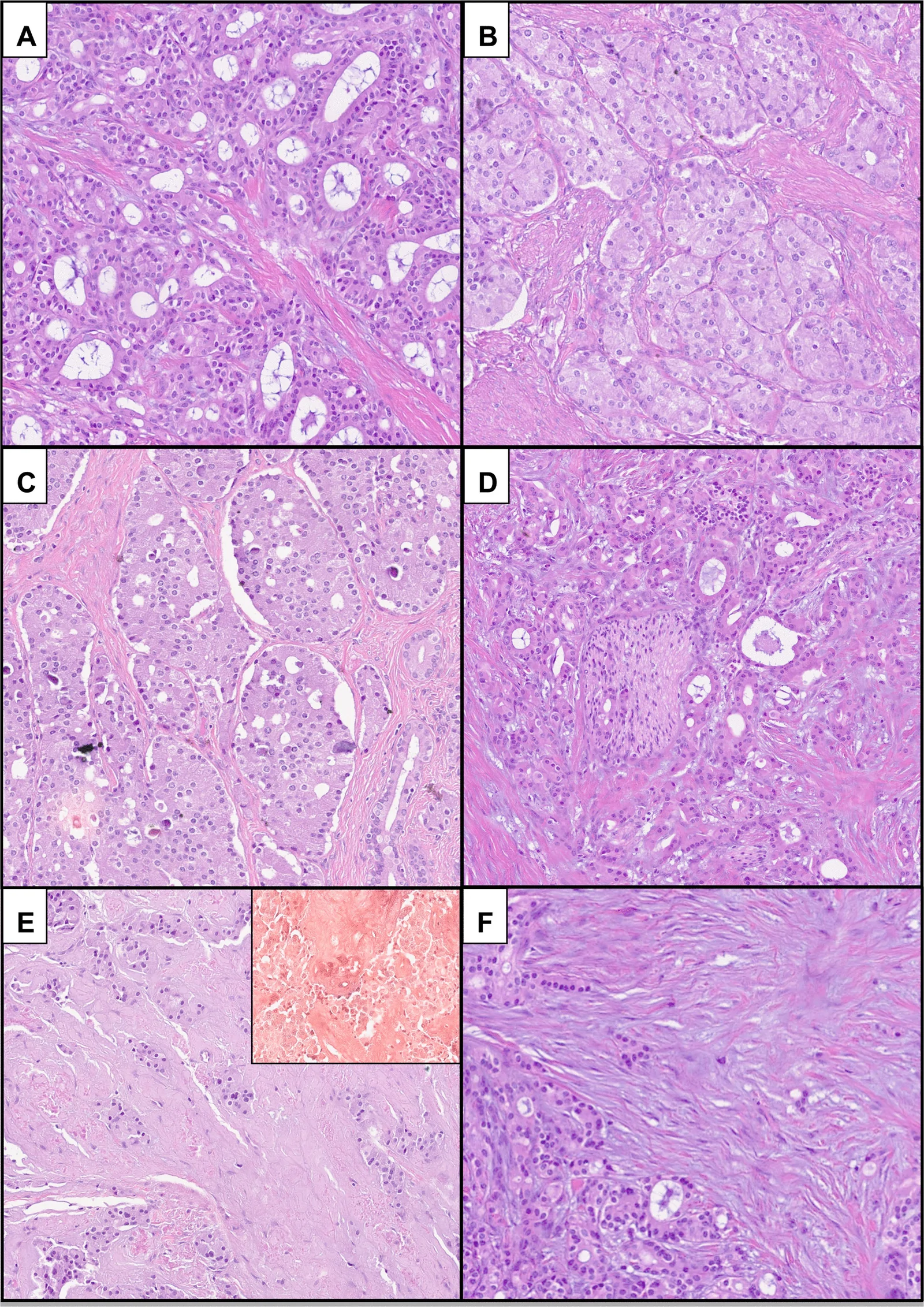
Histologic features of sporadic ampullary somatostatin-producing D cell neuroendocrine tumors (SAMSOM-NETs). **A** The typical tubulo-glandular pattern is seen. **B** Areas with a trabecular/nesting pattern may also be observed. **C** Cribriforming is prominent in some cases. **D** The tumor stroma is usually scant. Also note a perineural invasion by the SAMSOM-NET. **E** Some tumors show amyloid stroma. In the inset, Congo red staining of the same case, showing positivity of the stroma. **F** A single pretreated case features a fibrotic/desmoplastic stroma in the deep invasive areas of the tumor. (**A**-**F**, H&E)

Tumor cell cytoplasm was typically abundant and finely granular, while the nuclei were uniform with a granular chromatin pattern. Tubulo-glandular architecture was observed in all cases (Fig. 1A) and was classified as diffuse, substantial, and focal in 4 (37%), 5 (46%), and 2 (18%) cases, respectively. Trabecular and/or solid areas were also seen in all cases and represent the dominant tumor architecture in 4 tumors (Fig. 1B). Cribriform structures were observed in 8 tumors (Fig. 1C).

Although tumor stroma was scant in most cases (Fig. 1D) with very few or no immune cells, abundant Congo red-positive homogeneous eosinophilic stroma with apple green birefringence, consistent with amyloid stroma, was observed in 4 cases (36%) (Fig. 1E), while in a stage IV case who received everolimus before surgical resection (case #11), the stroma was more fibrotic/desmoplastic and surrounded single cells or short cords of tumor cells (Fig. 1F).

**Fig. 2:**
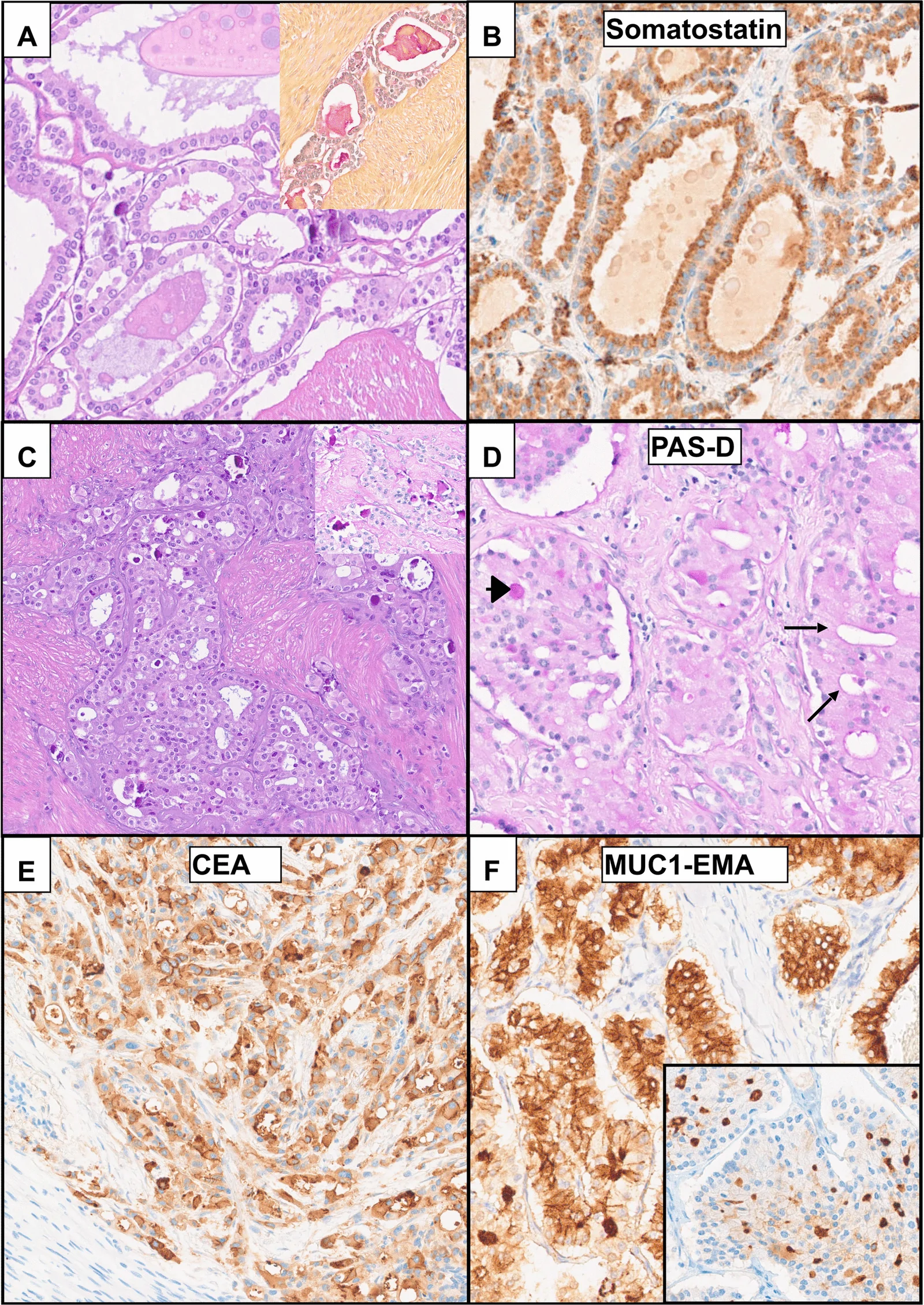
Amphicrine-like features in SAMSOM-NETs. **A** and **B**) A representative image of a tumor exhibiting thyroid follicular-like structures composed of cystically dilated glands containing eosinophilic colloid-like material, resembling thyroid macrofollicles (**A**). In the inset of (A), mucicarmine staining of the same case, exhibiting intraluminal positivity of the colloid-like material. Tumor cells lining the follicular structure are somatostatin immunoreactive (**B**). **C**) Intraluminal psammoma bodies are seen. Note, in the inset, PAS-D positivity of psammoma bodies of the same case. **D**) PAS-D positivity is seen both by apical tumor cell membranes (arrows) and intraluminal content of tubulo-glandular structures (arrowhead). **E**) Membranous and cytoplasmic CEA positivity in neoplastic cells may be observed. **F**) MUC1-EMA is consistently positive with variable membranous and cytoplasmic immunoreactivity. In some cases, the positivity is mostly intraluminal (see the inset). (A, C, H&; B, Somatostatin immunohistochemistry; D, inset of C, PAS-D histochemistry; E, CEA immunohistochemistry; F, MUC1-EMA immunohistochemistry)

Two (18%) SAMSOM-NETs showed thyroid follicular-like structures composed of cystically dilated glands containing eosinophilic PAS-positive colloid-like material, resembling thyroid macrofollicles; this material was also mucicarmine-positive (Fig. 2A and B). Psammoma bodies were seen in 7 (64%) cases and were numerous in 2 cases; they were almost always PAS-positive (Fig. 2C). PAS-positive intraluminal mucinous material was seen in 8 (73%) SAMSOM-NETs; PAS also stained the apical aspect of a few glandular structures in 5 cases (Fig. 2D).

### Immunohistochemical Findings

The immunohistochemical findings of 11 SAMSOM-NETs are reported in Table 2 (general neuroendocrine markers, cytokeratins, MUC1, CEA and S100) and Table 3 (hormones and transcription factors) and summarized below.

**Table 2:** Immunohistochemical data (general neuroendocrine markers, cytokeratins, MUC1, carcinoembryonic antigen and S100 protein) of the 11 sporadic ampullary somatostatin-producing D-cell neuroendocrine tumors examined

	Chromogranin-A	Synaptophysin	INSM1	SSTR2A	CK CAM 5.2	CK AE1-AE3	CK7	CK20	MUC1	mCEA	S100
#1	Pos (D, W)	Pos (D, S)	Pos (D, S)	Score 0	Pos (D, S, DOT-like 80%)	Pos (D, W, DOT-like 0%)	Neg	Neg	Pos (D, S)	Neg	Score 1 +
#2	Pos (D, S)	Pos (D, S)	Pos (D, W)	Score 0	Pos (D, S, DOT-like 50%)	Pos (D, S, DOT-like 60%)	Pos (D, S, DOT-like 20%)	Neg	Pos (D, S)	Neg	Score 3 +
#3	Pos (D, W)	Pos (D, S)	Pos (D, W)	Score 0	Pos (D, S, DOT-like 80%)	Pos (D, S, DOT-like 80%)	Pos (F, S, DOT-like 0%)	NE	Pos (D, S)	Neg	Score 3 +
#4	Pos (D, S)	Pos (D, S)	Pos (D, S)	Score 0	Pos (D, S, DOT-like 40%)	Pos (D, S, DOT-like 40%)	Pos (F, S, DOT-like 20%)	Neg	Pos (D, S)	Pos (F, S)	Score 3 +
#5	Pos (D, S)	Pos (D, S)	Pos (D, W)	Score 0	Pos (D, S, DOT-like 0%)	Pos (D, S, DOT-like 5%)	Pos (F, S, DOT-like 0%)	Neg	Pos (D, W)	Neg	Score 3 +
#6	Pos (D, W)	Pos (D, S)	Pos (D, W)	Score 0	Pos (D, S, DOT-like 2%)	Pos (D, S, DOT-like 5%)	Pos (D, S, DOT-like 0%)	Neg	Pos (D, S)	Pos (F, S)	Score 0
#7	Pos (D, W)	Pos (D, S)	Pos (D, W)	Score 0	Pos (D, S, DOT-like 80%)	Pos (D, S, DOT-like 80%)	Pos (D, S, DOT-like 15%)	Neg	Pos (D, S)	Neg	Score 3 +
#8	Pos (D, W)	Pos (D, S)	Pos (D, S)	Score 0	Pos (D, S, DOT-like 75%)	Pos (D, S, DOT-like 70%)	Pos (D, S, DOT-like 75%)	Neg	Pos (D, S)	Pos (F, S)	Score 2 +
#9	Pos (D, W)	Pos (D, S)	Pos (D, S)	Score 0	Pos (D, S, DOT-like 75%)	Pos (D, S, DOT-like 80%)	Pos (F, S, DOT-like 40%)	Neg	Pos (D, S)	Neg	Score 3 +
#10	Pos (D, S)	Pos (D, S)	Pos (D, S)	Score 0	Pos (D, S, DOT-like 40%)	Pos (D, S, DOT-like 50%)	Neg	Neg	Pos (D, S)	Pos (F, S)	Score 2 +
#11	Pos (D, W)	Pos (D, S)	Pos (D, S)	Score 0	Pos (D, S, DOT-like 30%)	Pos (D, S, DOT-like 20%)	Pos (D, S, DOT-like 30%)	Neg	Pos (D, S)	Pos (D, S)	Score 3 +

Legend: CK = Cytokeratin; D = Diffuse; F = Focal; mCEA = Monoclonal carcinoembryonic antigen; Neg = Negative; NE = Not Evaluable; Pos = Positive; S = Strong; SSTR2A = Somatostatin Receptor Type 2 A, W = Weak

**Table 3:** Immunohistochemical data (hormones and transcription factors) of the 11 sporadic ampullary somatostatin-producing D-cell neuroendocrine tumors examined

	Somatostatin*	Gastrin	Serotonin	PP	Glucagon	Insulin	Calcitonin	ISL1	PDX1	ARX	CDX2	SATB2	TTF-1	ER	PR
#1	Pos (D, 100%, S)	Neg	Neg	Neg	Neg	Neg	Neg	Pos (D, S)	Pos (D, W)	Neg	Neg	Neg	Neg	Neg	Neg
#2	Pos (D, 100%, S)	Neg	Neg	Neg	Neg	Neg	Neg	Pos (D, S)	Pos (D, W)	Neg	Neg	Neg	Neg	Neg	Neg
#3	Pos (D, 100%, S)	Neg	Neg	Neg	Neg	Neg	Neg	Pos (D, S)	Pos (D, W)	Pos (F, S)	Neg	Neg	NV	Neg	Neg
#4	Pos (D, 100%, S)	Pos (F, S)	Neg	Neg	Neg	Neg	Neg	Pos (D, S)	Pos (D, S)	Pos (F, S)	Pos (F, W)	Neg	Neg	Neg	Neg
#5	Pos (D, 70%, S)	Pos (F, S)	Neg	Neg	Neg	Neg	Neg	Pos (D, S)	Pos (D, S)	Neg	Pos (D, W)	Neg	Neg	Neg	Neg
#6	Pos (D, 100%, S)	Pos (F, S)	Neg	Pos (F, S)	Neg	Neg	Neg	Pos (D, S)	Pos (D, S)	Neg	Pos (D, W)	Neg	Neg	Neg	Neg
#7	Pos (D, 100%, S)	Pos (F, S)	Neg	Neg	Neg	Neg	Neg	Pos (D, S)	Pos (D, W)	Neg	Neg	Neg	Neg	Neg	Neg
#8	Pos (D, 100%, S)	Pos (F, S)	Pos (F, S)	Pos (F, S)	Neg	Neg	Neg	Pos (D, S)	Pos (D, S)	Pos (F, S)	Pos (D, W)	Neg	Neg	Neg	Pos (F, S)
#9	Pos (D, 100%, S)	Neg	Neg	Neg	Neg	Neg	Neg	Pos (D, S)	Pos (D, W)	Neg	Neg	Neg	Neg	Neg	Neg
#10	Pos (D, 100%, S)	Pos (F, S)	Neg	Neg	Neg	Neg	Neg	Pos (D, S)	Pos (D, S)	Neg	Pos (F, W)	Neg	Neg	Pos (F, S)	Neg
#11	Pos (D, 100%, S)	Pos (F, S)	Neg	Pos (F, S)	Neg	Neg	Neg	Pos (D, S)	Pos (D, S)	Pos (F, S)	Pos (D, S)	Neg	Neg	Neg	Pos (F, S)

D = Diffuse; ER = Estrogen receptor; F = Focal; NV = Not valuable; PP = Pancreatic Polypeptide; PR = Progesterone receptor; S = Strong; W = Weak. *the percentage of neoplastic cells expressing somatostatin is indicated

**Fig. 3:**
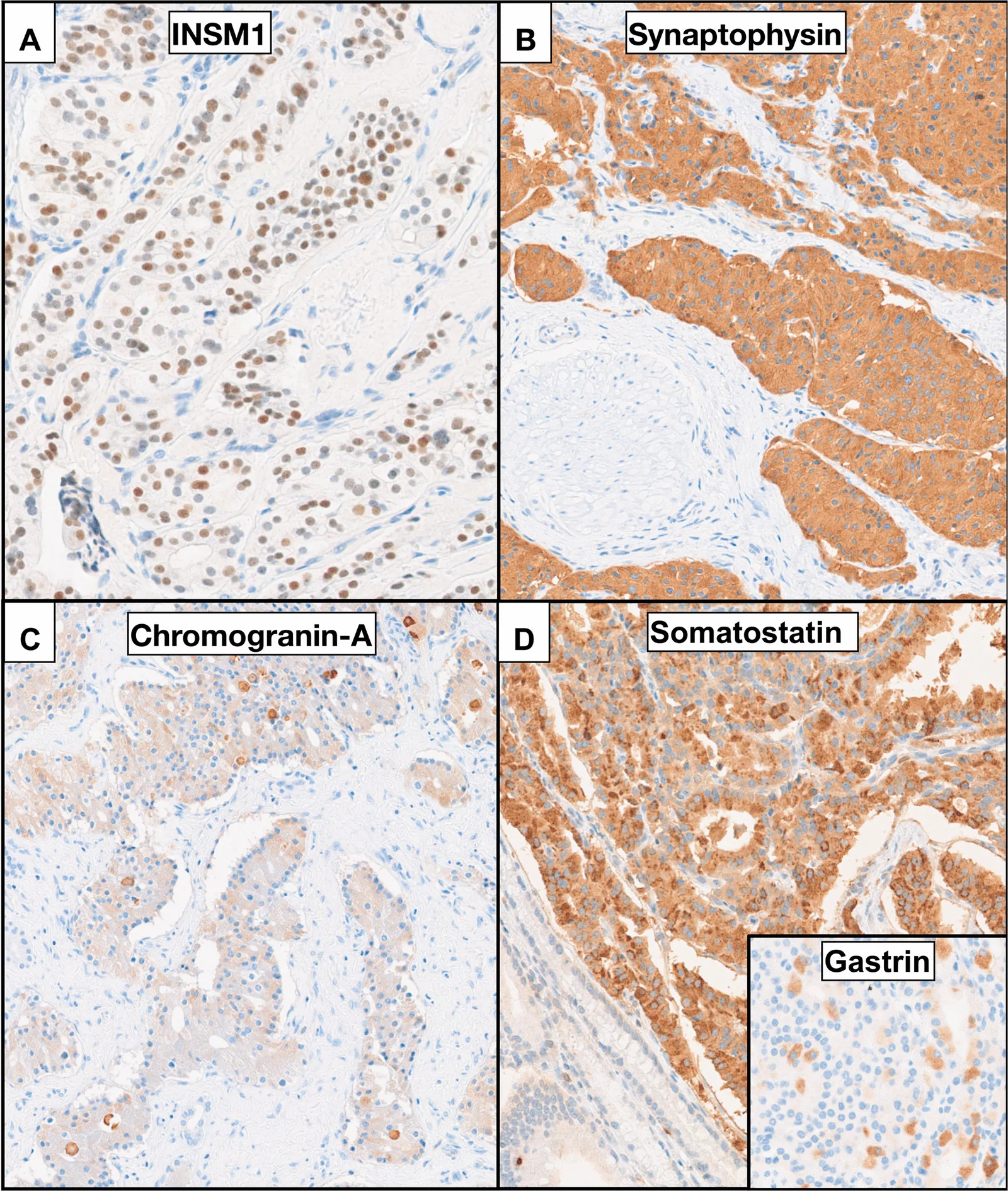
Neuroendocrine marker and hormone expression in SAMSOM-NETs. **A**-**C**) Tumor cells of SAMSOM-NETs show strong and diffuse positivity for INSM-1 (**A**) and synaptophysin (**B**), whereas chromogranin-A is usually diffuse but weak in tumor cells and also often positive in psammoma bodies (**C**). **D**) SAMSOM-NETs express somatostatin strongly and diffusely, while other hormones such as gastrin are focally positive in a few cases (inset of D). (**A** INSM-1 immunohistochemistry; **B** synaptophysin immunohistochemistry; **C** chromogranin-A immunohistochemistry; **D** somatostatin immunohistochemistry; inset of D, gastrin immunohistochemistry)

INSM1 was diffusely positive in all tumors (Fig. 3A). Synaptophysin was strongly and diffusely positive in all tumors (Fig. 3B). Chromogranin A was diffusely positive in all but one tumor, often with weak intensity (Fig. 3C). SSTR2A membranous expression was scored 0 (negative) in all cases.

**Fig. 4:**
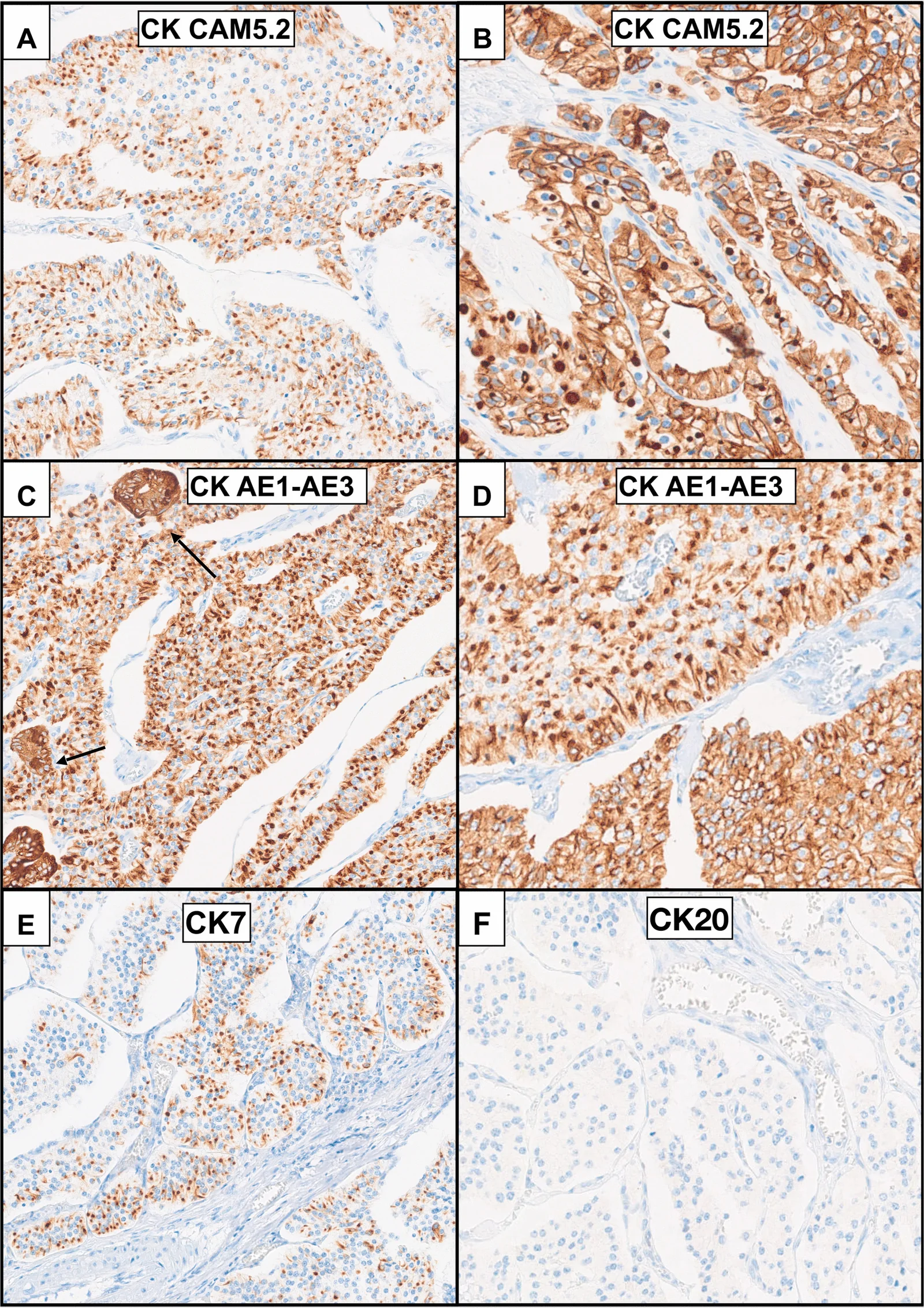
Cytokeratin expression in SAMSOM-NETs. **A**-**E**) SAMSOM-NETs showing dot-like positivity for cytokeratin (CK) CAM5.2 (**A**, **B**), CK AE1-AE3 (**C**, **D**) and CK7 (**E**). Note in (**C**) ampullary ductules (arrows) for comparison. In some tumor areas (**B**, **D**) both membranous and dot-like CK positivity is seen. In a few cases, CK CAM5.2 and CK AE1-AE3 dot-like positivity in > 70% of tumor cells are identified. **F**) CK20 is consistently negative. (**A**, **B** CK CAM5.2 immunohistochemistry; **C**, **D** CK AE1-AE3 immunohistochemistry; **E** CK7 immunohistochemistry; **F** CK20 immunohistochemistry)

CK CAM5.2 and AE1/AE3 were diffusely positive in all cases (Fig. 4A-D). All SAMSOM-NETs showed dot-like cytoplasmic reactivity for either CK CAM5.2 or CK AE1/AE3. Paranuclear dot-like positivity in > 70% of tumor cells was observed in 5 cases with CK CAM5.2 and 4 cases with CK AE1/AE3. In all SAMSOM-NETs, a membranous/cytoplasmic CK positivity was also observed in a variable percentage of cells. Paranuclear dot-like expression of CK CAM5.2 was more frequent in SAMSOM-NETs (91%) compared to our series of Pan-NETs (3 out of 20 cases, 15%, *p* < 0.001). CK7 was positive in 82% of SAMSOM-NETs, with dot-like reactivity in six of nine positive cases (Fig. 4E). CK20 was negative in all tested tumors (Fig. 4F).

CEA was expressed in 5 (45%) SAMSOM-NETs, with focal luminal and cytoplasmic reactivity in 4 cases, and strong and diffuse (70% of tumor cells) reactivity in one tumor showing follicular-like structures with intraluminal mucins (Fig. 2E). Apomucin MUC1 was diffusely expressed in all tumors, mostly (9 cases) with a combined apical/luminal and cytoplasmic positivity (Fig. 2F). In addition, intraluminal content and psammoma bodies typically showed MUC1 reactivity. The secretory gel-forming mucins tested (MUC2, MUC5AC, and MUC6) were consistently negative. The pancreatic acinar cell marker BCL-10 was completely negative in 10 cases investigated, while one case exhibited equivocal (weak) positivity in rare tumor cells.

In addition to somatostatin, which was strongly and diffusely expressed in all tumors (Fig. 3D), SAMSOM-NETs may express other hormones, including gastrin (64%) (Fig. 3D, inset), pancreatic polypeptide (27%), calcitonin (27%), and serotonin (9%). Seven cases (64%) expressed more than one hormone; however, in all cases, the expression of hormones other than somatostatin was focal. Insulin and glucagon were negative in all tumors. In the 4 cases with amyloid stroma somatostatin stained the amyloid deposits.

**Fig. 5:**
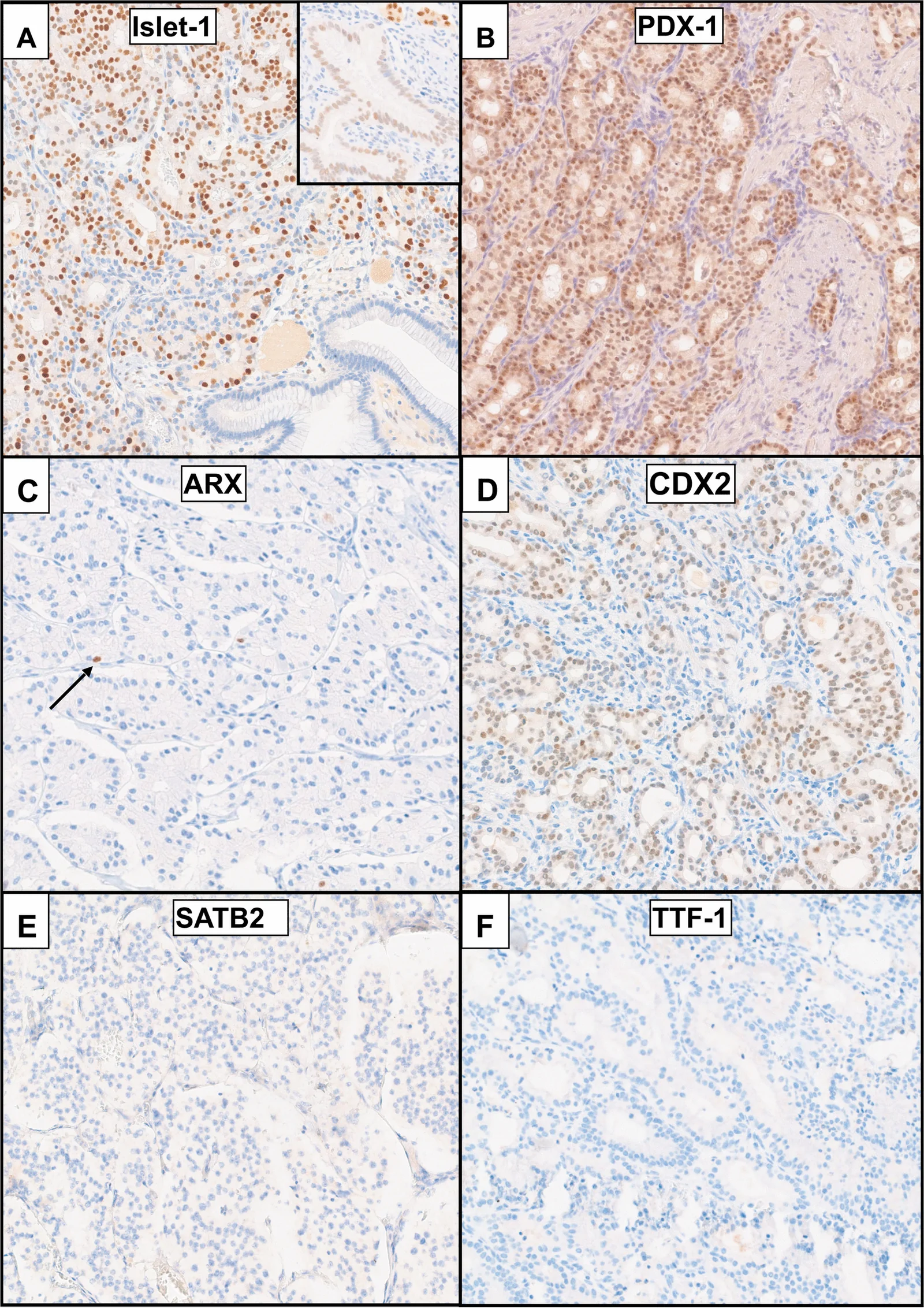
Transcription factors expression of SAMSOM-NETs. Tumor cells exhibit strong and diffuse positivity for ISL1 (**A**), which may also show weak positivity in ampullary ducts (inset of A), as well as for PDX1 (**B**). ARX is negative, or positive in rare cells (arrow)(**C**). CDX2 shows weak and diffuse immunoreactivity in this case, although its expression is variable (**D**). SATB2 (**E**) and TTF-1 (**F**) are consistently negative. (**A** ISL1 immunohistochemistry; **B** PDX1 immunohistochemistry; **C** ARX immunohistochemistry; **D** CDX2 immunohistochemistry; **E** SATB2 immunohistochemistry; **F** TTF-1 immunohistochemistry)

ISL1 was strongly and diffusely positive in all tumors (Fig. 5A). PDX1 was diffusely positive in all tumors (Fig. 5B). ARX was only focally positive in 4 (36%) tumors (Fig. 5C). Focal gastrin expression was observed in three of four cases with focal ARX expression. CDX2 was positive in 6 (55%) tumors, most showing weak expression (Fig. 5D). SATB2 and TTF-1 were consistently negative (Fig. 5E and F). Estrogen and progesterone receptors were focally expressed in 1 and 2 tumors, respectively.

**Fig. 6:**
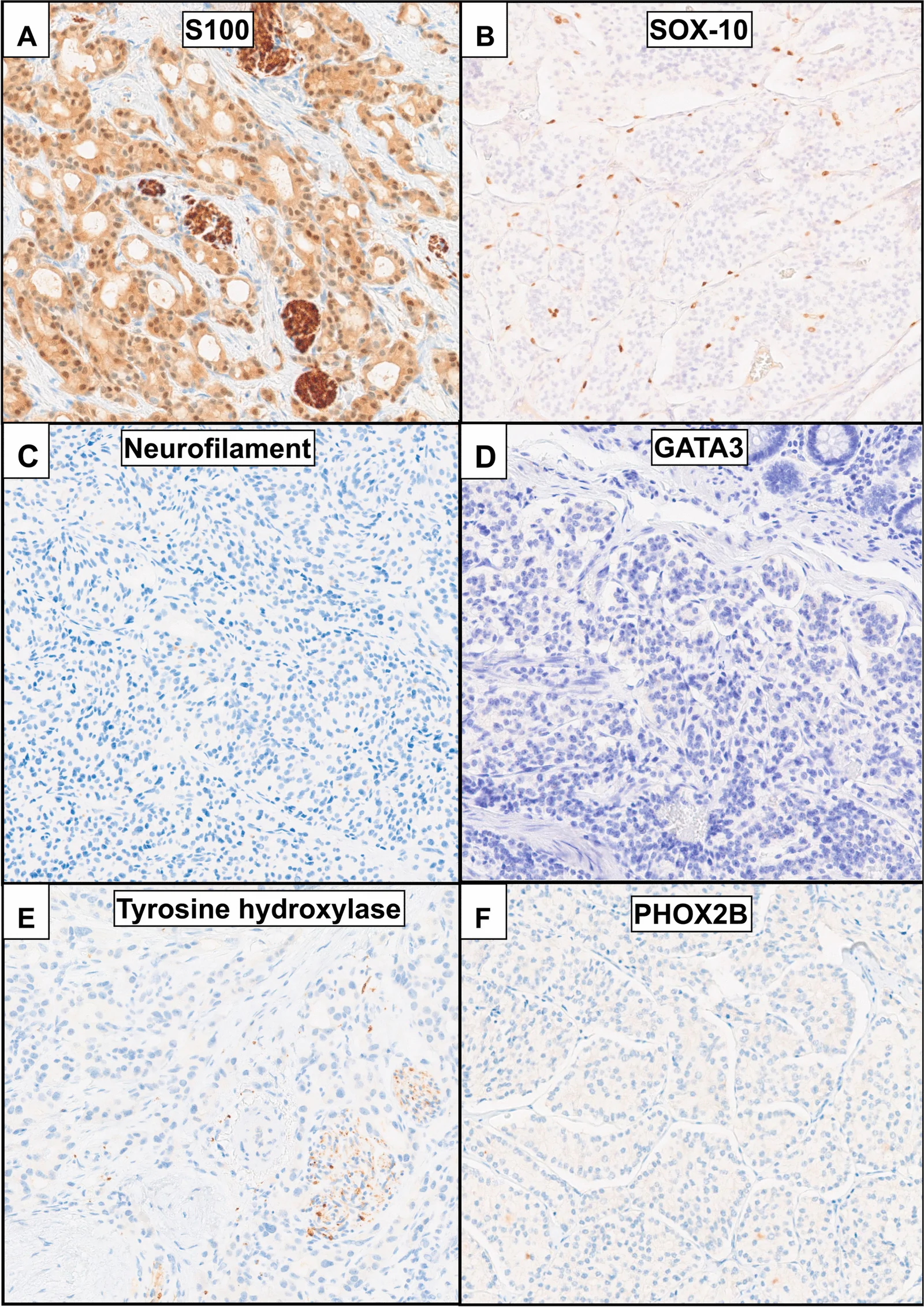
Neural crest and sympatico-adrenal marker expression in SAMSOM-NETs. **A**) Tumor cells are diffusely positive for S100; note the strong S100 reactivity of nerve structures invaded by the tumor. **B**) Tumor cells are negative for SOX10, while SOX10-positive sustentacular cells may be present, surrounding tumor cell nests mimicking a paraganglioma. **C**) Neurofilament protein is not expressed. **D** and **F**) Sympatico-adrenal markers GATA3, tyrosine hydroxylase (TH), PHOX2B are consistently negative. Note, as control for TH (**E**), the positivity of neural elements. (**A** S100 immunohistochemistry; **B** SOX10 immunohistochemistry; **C** Neurofilament immunohistochemistry; **D** GATA3 immunohistochemistry; **E** TH immunohistochemistry; **F** PHOX2B immunohistochemistry)

S100 protein was positive in tumor cells in all but one case, whereas SOX10 was negative in tumor cells in all cases (Fig. 6A, B). S100 and SOX10 positive sustentacular cells were found in 4 cases. Neurofilament protein and sympatico-adrenal markers GATA3, TH, PHOX2B were negative in all cases (Fig. 6C-F).

SDHB expression was retained in all tumors and ∝ -inhibin was consistently negative (Supplementary Fig. 1A, B). β-catenin exhibited membranous score 3 + positivity in all cases, with focal nuclear positivity in 3 cases (Supplementary Fig. 1C). E-cadherin membranous expression was retained (score 3 + in 9 cases, score 2 + in 2 cases) in all cases (Supplementary Fig. 1D). Notably, in case #11, E-cadherin membranous expression was incomplete in the most deep and desmoplastic tumor areas.

### Molecular Findings

According to the QC performed before library preparation, all samples showed highly fragmented DNA and generally low RNA quality, as expected for archival FFPE tissues collected between 2007 and 2022. Nevertheless, libraries were successfully prepared and sequenced, and sequencing performance was consistent across cases.

All detected genomic alterations, including pathogenic or likely pathogenic SNVs, splice-site alterations, insertions/deletions (indels), gene fusion transcripts, as well as MSI status and TMB, are reported in Table 4. No pathogenic or likely pathogenic CNVs were identified in the analyzed cohort. Exploratory LOH assessment did not identify any pathogenic or likely pathogenic variants meeting the predefined criteria for LOH compatibility (VAF ≥ 60%). In 7 out of 11 tumors (64%), at least one genetic alteration was detected, whereas 4 cases did not show any pathogenic alterations in the genes investigated.

**Table 4:** Molecular data: genomic alterations (SNVs, Indels, fusion transcripts, MSI status, TMB) with clinical or potential clinical significance

Case Number	Neoplastic cellularity	SNVs, Indels				Fusion transcripts		MSI status		TMB	
		Variant type	Gene and transcript	cDNA change and Protein change	VAF	Fusion gene and breakpoints	Fraction	Unstable loci	MSI status	N mutations/Mb	TMB status
#1	80%	Missense	*HRAS* (ENST00000311189)	c.182A > G p.(Gln61Arg)	70.0%	None		NA	Undetermined	NA	Undetermined
#2	80%	Missense	*HRAS* (ENST00000311189)	c.181C > A p.(Gln61Lys)	37.0%	NTRK3::PRDM4 E14::E6	40.0%	5.8%	MSS	0	Low
#3	70%	None				None		0	MSS	NA	Undetermined
#4	40%	None				None		6.3%	MSS	NA	Undetermined
#5	70%	None				None		1.4%	MSS	0	Low
#6	80%	None				None		0	MSS	NA	Undetermined
#7	80%	Splice donor	*CDK12* (ENST00000447079)	c.2963 + 1G > A p.?	66.7%	None		0	MSS	NA	Undetermined
#8	60%	Missense	*HRAS* (ENST00000311189)	c.37G > C p.(Gly13Arg)	33.9%	None		5.4%	MSS	NA	Undetermined
#9	80%	Missense	*HRAS* (ENST00000311189)	c.182A > G p.(Gln61Arg)	35.4%	None		3.9%	MSS	0	Low
#10	80%	Splice acceptor	*NF1* (ENST00000358273)	c.61-2A > T p.?	37.7%	None		8.1%	MSS	0.9	Low
		Missense	*NF1* (ENST00000358273)	c.2842_2843delCAinsT p.(Gln948fs)	33.7%						
#11	70%	Missense	*KRAS* (ENST00000256078)	c.34G > C p.(Gly12Arg)	25.7%	None		7.5%	MSS	2.6	Low

Indels = Insertions/deletions; MSI = MicroSatellite Instability; MSS = MicroSatellite Stable; N = Number; NA = Not Assessable; SNVs = Single Nucleotide Variants; TMB = Tumor Mutational Burden; VAF = Variant Allele Frequency

The median number of genetic alterations *per* tumor was 1 (range: 0–2). Only two cases harbored more than one genomic alteration. Case #2 harbored both an *HRAS* variant (VAF: 37%) and an *NTRK3*::*PRDM4* fusion (fraction: 40%); pan-TRK immunostaining was negative in this tumor. Case #10 showed two different *NF1* gene alterations (1 frameshift variant and 1 splice-site alteration, VAF 33.7% and 37.7% respectively), which were not detected in the non-neoplastic intestinal tissue from the same patient, confirming their somatic nature. *HRAS* was the only recurrently mutated gene, observed in 4 SAMSOM-NETs (36%). All *HRAS* alterations were missense SNVs with VAFs ranging from 33.9% to 70%. Predicted protein changes were p.Gln61 [3 cases, 2/3: p.(Gln61Arg); 1/3:p.(Gln61Lys)] and p.(Gly13Arg) (1 case). All cases with *HRAS* SNVs arose in the major ampulla. A *KRAS* missense variant [p.(Gly12Arg), VAF: 25%] was found in an SAMSOM-NET of the minor ampulla (case #11), whereas a splice-site alteration in the *CDK12* gene was detected in a tumor of the major ampulla (case #7).

Somatic pathogenetic or likely pathogenetic SNVs in the RAS pathway (including *HRAS, KRAS*, and *NF1*) and in the kinase-associated pathways (also including the *CDK12* mutated case), were identified in 6 (54%) and in 7 (64%) out of 11 SAMSOM-NETs, respectively (Table 4).

Tumors harboring RAS pathway alterations were significantly larger than those without such alterations (median size, 33 mm versus 15 mm; Mann–Whitney U test, p = 0.004). No statistically significant differences were identified between tumors with and without RAS pathway alterations for other clinicopathologic parameters. Similarly, no significant differences were observed when comparing the broader subgroup of cases harboring SNVs in kinase signaling pathways (including RAS pathway alterations and *CDK12* mutations) with the remaining SAMSOM-NETs. Compared with tumors lacking RAS pathway alterations, RAS pathway-altered SAMSOM-NETs occurred in older patients (median age, 65 versus 58 years), more frequently involved the major ampulla (83% versus 40%), and showed larger mean and median tumor sizes (32.5 mm and 33 mm versus 15.6 mm and 15 mm, respectively). RAS pathway-altered tumors also showed higher frequencies of lymphatic invasion (100% versus 60%), perineural invasion (50% versus 20%), and pT3 stage (50% versus 20%). Systematic lymphadenectomy was performed more frequently in this subgroup (5/6, 83%). Mono-hormonality (defined as the absence of expression of hormones other than somatostatin) and psammoma bodies were also more frequently observed among RAS pathway-altered tumors compared with cases lacking RAS pathway alterations (50% versus 20% and 83% versus 40%, respectively). All three patients who developed distant metastases harbored RAS pathway alterations (one *HRAS*, one *KRAS*, and one *NF1* alteration). Given the limited sample size, these observations should be considered descriptive and hypothesis-generating.

All evaluable tumors (*n* = 10 for MSI status and *n* = 5 for TMB) were found to be microsatellite stable (MSS; unstable loci ranging from 0 to 8.1%) and to have a low TMB (range: 0–2.6 mutations/Mb).

### Follow-up Data

Patients were followed for a median of 104 months (range: 35–222 months). Eight patients without distant metastases were alive with no evidence of disease after a median follow-up of 128 months. Among the three patients presenting with liver metastases at diagnosis, one died of unrelated causes 46 months after surgery, while the remaining two were alive with disease at 36 and 104 months, respectively, following resection of the primary tumor.

## Discussion

Our study represents the first comprehensive attempt to characterize SAMSOM-NETs from histological, histochemical, immunohistochemical, and molecular perspectives. The findings provide original insights that contribute to a deeper understanding of these NETs, showing distinctive CK expression patterns and their peculiar genomic profile with recurrent RAS mutation.

From a clinico-pathological standpoint, our study confirms that SAMSOM-NETs are non-functioning tumors that clinically present with symptoms related to biliary obstruction (e.g., jaundice), chronic gastrointestinal bleeding, or nonspecific symptoms such as abdominal pain in adult patients with a male predominance [4, 22]. Although SAMSOM-NETs are well-differentiated neoplasms, they appear to display a significant metastatic potential, with a high frequency of lymph node and/or distant metastases observed either at diagnosis or during follow-up. Notably, all cases in our series in which lymph nodes were evaluated showed nodal metastases, and liver metastases were identified in three cases. Despite this, the histological grade in our cohort was G1 in the majority of cases (64%), while the remaining cases (36%) were G2, with a Ki-67 index typically < 10%. Furthermore, tumor necrosis was not observed in any case. Of note, Garbrecht et al. [4] reported two SAMSOM-NETs with a Ki-67 index > 20% (which would be now classified as NET G3). Despite their highly invasive and metastatic potential, prognosis appears to be favorable. Indeed, no tumor-related deaths were observed in our series over a relatively long follow-up period (median follow-up: 104 months), a finding consistent with the study by Garbrecht et al. [4]. This behavior is consistent with that observed in NETs arising from other sites, particularly non-functioning Pan-NETs, in which patients often experience prolonged survival even after the diagnosis of metastases [12].

SAMSOM-NETs are tumors that exhibit diffuse expression of general neuroendocrine markers, most notably synaptophysin and INSM1, supporting their neuroendocrine nature, despite weak and occasionally focal positivity for chromogranin A. In contrast to most pancreatic and non-ampullary duodenal NETs [9, 12], all SAMSOM-NETs in our series lacked SSTR2A expression, as also previously suggested [9]. The biological basis of the absent SSTR2A expression observed in SAMSOM-NETs remains unclear. Differences in lineage-specific differentiation programs and neuroendocrine cell phenotypes may contribute to this finding, as SSTR2 expression has been shown to vary among gastrointestinal neuroendocrine cell populations, with D cells and enterochromaffin (EC) cells of the stomach, duodenum, and rectum exhibiting lower expression levels compared with other neuroendocrine cell types [23]. If validated in larger independent cohorts, SSTR2A negativity could represent a useful ancillary feature for distinguishing ampullary somatostatin-producing NETs from pancreatic and non-ampullary duodenal NETs in biopsy specimens, although it should not be considered a standalone diagnostic marker. This immunohistochemical finding may also have clinical diagnostic implications, as some of these NETs may not be detectable by somatostatin receptor–based nuclear medicine imaging (e.g., ^^68^ Ga-DOTATOC PET/CT), as recently described in a case report of an ampullary somatostatin-producing NET associated with NF1 syndrome [24]. These tumors exhibit morphological and immunohistochemical features that may mimic well-differentiated adenocarcinomas, the main differential diagnosis with important clinical implications. Common findings included tubuloglandular and/or cribriform architecture (100%), intraluminal or apical PAS-D–positive material (73%), occasional thyroid follicle-like structures containing PAS- and mucicarmine-positive colloid-like material (18%), MUC1 expression (100%), and mCEA positivity in a substantial subset of cases (45%). These features have been variably described in SAMSOM-NETs in several previous studies [2, 4, 9, 22, 25]. Nevertheless, no exocrine or amphicrine features were identified in SAMSOM-NETs, as evidenced by the morphologic and histochemical absence of intracytoplasmic mucin and the lack of expression of tested secretory mucins (MUC2, MUC5AC, and MUC6) and the acinar cell marker BCL10. Therefore, although SAMSOM-NETs may show amphicrine-like features, they must not be classified as true amphicrine neoplasms, amphicrine-like carcinomas, or MiNENs [12]. Psammoma bodies, frequently present in NF1-associated ampullary NETs, were also commonly encountered in SAMSOM-NETs (63.6%) in our series. They were often located within glandular lumina and frequently exhibited PAS-D and MUC1 positivity. Although the pathogenesis of psammoma bodies in SAMSOM-NETs remains incompletely understood, these findings may suggest a contributory role of PAS-positive material and the MUC1 in their formation and growth, complementing the hypothesis based on cellular degeneration/necrosis leading to the development of intracytoplasmic calcific bodies [17, 26].

Furthermore, the frequent expression of S100 protein in tumor cells and/or sustentacular cells, together with SOX10 positivity in sustentacular cells in a subset of cases, may pose diagnostic challenges in the differential diagnosis with paragangliomas and composite gangliocytoma/neuroma and neuroendocrine tumors (CoGNETs) that share with SAMSOM-NETs the expression of general neuroendocrine markers and, in some cases, somatostatin expression [19, 27]. SAMSOM-NETs can be distinguished from paragangliomas by their expression of CKs and by the absence of markers of sympathetic/autonomic differentiation (GATA3, PHOX2B, and TH), while SAMSOM-NETs lack the characteristic triphasic morphology typical of CoGNETs (i.e., absence of a gangliocytoma/ganglioneuroma component) and are negative for PR and negative, or only focally positive, for PP, markers typically expressed in the epithelial component of CoGNETs [28]. Additionally, CoGNETs have been recently reported to express ARX [17, 29], which is negative or only focally positive in a very small subset of cells in the SAMSOM-NETs analyzed in this study.

Similarly to other NETs, such as pancreatic insulinomas, medullary thyroid carcinoma and lactotroph pituitary NETs (PitNETs) [27], 4 cases (36%) of SAMSOM-NETs in our cohort exhibited Congo red–positive amyloid stroma, showing apple-green birefringence under polarized light, and were positive for somatostatin. Somatostatin-derived amyloid deposition associated with duodenal/ampullary NETs has recently been described; in these studies, somatostatin was identified as the predominant constituent of the amyloid deposits, based on the results of immunohistochemical and proteomics analyses [30, 31]. While the stroma was scant and fibrovascular in most of the remaining cases, a single case showed a fibrous-desmoplastic stroma within the deeply invasive component, possibly reflecting, at least in part, the effects of preoperative therapy with an mTOR inhibitor (i.e., everolimus). Notably, this same case exhibited diffuse expression of mCEA and MUC1, underscoring that the differential diagnosis between SAMSOM-NET and ampullary adenocarcinoma can occasionally be challenging and may require the application of broad immunohistochemical panels, including general neuroendocrine markers and somatostatin.

An interesting and previously unreported finding in SAMSOM-NETs is the frequent paranuclear dot-like expression of CK CAM5.2, CKAE1/AE3, and CK7, observed in 91%, 91%, and 54% of cases, respectively, typically associated with variable membranous cytokeratins expression. This CK pattern has been described in other neuroendocrine neoplasms, such as Merkel cell carcinoma, small cell neuroendocrine carcinomas, specific subtypes of PitNETs, middle ear NETs, cauda equina NETs, and, albeit less frequently, Pan-NETs, as also confirmed in our study, particularly in VIPomas [27, 32–37]. These CK-positive cytoplasmic inclusions, corresponding ultrastructurally to juxtanuclear aggresomes of keratin filaments, have been variably referred to as “fibrous bodies,” “rhabdoid inclusions,” or “cytokeratin aggresomes.” Similar bundles and nodular aggregates of paranuclear microfilaments have also been previously described at the ultrastructural level in duodenal “somatostatinomas” [38]. In some cases, more than 70% of tumor cells exhibited this feature, as also reported in sparsely granulated somatotroph PitNETs [20, 39]. It is conceivable that the presence of such CK aggresomes may influence tumor cell biology by altering cytoplasmic architecture. In particular, they may impair secretory granule transport or exocytosis, potentially contributing to the typically non-functioning clinical behavior of ampullary somatostatin-producing NETs. Furthermore, they may reflect a less differentiated cellular phenotype, possibly contributing to the higher invasive and/or metastatic biological behavior of these tumors compared with gastrin cell NETs [5]. Whether this feature may have diagnostic value in histological subtyping of duodeno-ampullary NETs remains to be clarified in future studies. An additional noteworthy finding of our study is the frequent CK7 positivity (82% of cases) in SAMSOM-NETs, previously reported [9], together with their consistent CK20 negativity. CK7 expression is rare in gastrointestinal and Pan-NETs (< 10% of cases). On the other hand, it has been reported as positive in approximately two-thirds of pulmonary NETs [40], and has been variably described in middle ear NETs, with preferential expression in areas showing tubular architecture [41]. CK7 expression, together with the amphicrine-like features of SAMSOM-NETs, may represent an additional diagnostic pitfall in the differential diagnosis between SAMSOM-NETs and ampullary adenocarcinomas.

SAMSOM-NETs express ISL1 and PDX1 and often show weak CDX2 positivity, while they are negative or only focally positive for the other transcription factors tested. The expression of ISL1 and PDX1 has already been described in duodeno-ampullary NETs, including somatostatin-producing NETs; however, it is not specific to this anatomical subset, nor does it appear to be associated with particular hormonal subtypes, as it has also been reported in gastrin-producing duodenal NETs [42]. The transcription factor CDX2, which controls intestinal differentiation, also appears to be frequently expressed in SAMSOM-NETs (55%), albeit often weakly, while, in contrast to ISL1 and PDX1, CDX2 is only rarely expressed (approximately 10%) in Pan-NETs [43]. Nevertheless, CDX2 does not seem to be specific to this tumor subtype, as Delfin et al. reported CDX2 expression in 65% of duodenal NETs with no apparent association with hormone expression [17]. Conversely, Hermann et al. did not observe CDX2 expression in the small number of somatostatin-expressing duodenal NETs analyzed in their study; this discrepancy may be attributable to the use of different antibody clones in the two studies. Of note, SAMSOM-NETs in our series were negative for ARX, a transcription factor frequently expressed in Pan-NETs, where it is strongly associated with “alpha-cell-like” tumors and defines the A1 and A2 transcription factor-based subtypes [43]. ARX expression has also been reported in neuroendocrine cells of the normal duodenal mucosa and in duodenal NETs in a variable proportion of cases between 33 and 59% [17, 44, 45], without a clear association with hormone-based tumor subtypes. However, at least in murine models and in human stem cell–derived intestinal organoids, ARX appears to play a role in enteroendocrine cell subtype specification. Indeed, with ARX inactivation, enteroendocrine progenitors are reallocated into somatostatin-expressing cells, with a concomitant reduction in cholecystokinin, gastrin, secretin, and glucagon-like peptide-1 (GLP-1) producing cells [46, 47]. The typical absence of ARX expression in SAMSOM-NETs is consistent with phenotypic similarities to duodenal D cells [48] and may suggest a putative cell of origin within the progenitor or mature D-cell compartment of the duodenal/ampullary epithelium, although endocrine lineage relationships in the duodenum remain incompletely defined. PAX4 and/or HHEX expression would have further informed this interpretation; however, these antibodies were not included in the immunohistochemical panel used in the present analysis, and PAX4 has recently been reported to be negative in duodenal NETs, including those producing somatostatin [17]. Moreover, as approximately 60% of SAMSOM-NETs may also show focal expression of additional hormones, such as gastrin, calcitonin or PP, the possibility that these tumors arise from multihormonal precursor cells cannot be entirely excluded. Definitive confirmation of the cell of origin will require lineage-tracing approaches, single-cell transcriptomic analyses, and epigenetic profiling demonstrating a direct developmental relationship with normal D cells or their progenitors.

Comprehensive genomic profiling performed in our study demonstrated that SAMSOM-NETs are MSS tumors with a low TMB. In addition, neither recurrent pathogenic copy number alterations nor LOH-compatible events were identified, suggesting that large-scale genomic alterations are unlikely to represent a major molecular feature of these tumors. In addition, recurrent variants in the *HRAS* gene were identified in 36% of the analyzed SAMSOM-NETs. *HRAS* alterations have been described in other solid malignancies, both epithelial, including low-grade non-invasive papillary urothelial carcinoma, follicular thyroid carcinoma, the invasive encapsulated follicular variant of papillary thyroid carcinoma, medullary thyroid carcinoma, and squamous cell carcinomas of the head and neck, and non-epithelial, including paragangliomas/pheochromocytomas and melanomas [27]. Interestingly, one of our SAMSOM-NET cases also exhibited multiple somatic alterations of the *NF1* gene. In a subset of duodenal NETs associated with NF1 syndrome, somatic inactivation of the wild-type allele of the *NF1* gene has been reported [10]. Therefore, *NF1* alterations do not appear to be exclusive to NF1-associated NETs. Considering an additional case with *KRAS* mutation, overall alterations in the RAS pathway were identified in 54% of our SAMSOM-NETs cases. The presence of these alterations tended to be associated with a higher frequency of psammoma bodies and a lower frequency of expression of hormones other than somatostatin. Notably, all three cases with liver metastases harbored alterations in the RAS pathway. Although this observation is based on only three cases and does not establish an association, it raises the possibility that RAS-pathway dysregulation may contribute to hematogenous metastatic progression. However, the descriptive association between RAS pathway alterations and clinicopathologic features suggestive of more advanced disease should be interpreted with caution. Tumors harboring RAS pathway alterations were larger than those without such alterations, and tumor size is itself associated with a higher likelihood of local invasion, more extensive surgical treatment, and lymph node assessment. Accordingly, the higher frequency of pT3 stage, lymphatic and perineural invasion, and systematic lymphadenectomy observed in the RAS pathway-altered subgroup may be at least partly explained by differences in tumor size rather than by an independent biological effect of the molecular alterations. Given the limited sample size, these findings should be regarded as exploratory, and larger studies will be required to determine whether RAS pathway alterations independently correlate with clinicopathologic features after accounting for tumor size and stage. Of note, RAS mutations are generally rare in gastrointestinal and pancreatic NETs [49, 50]. Conversely, *KRAS* alterations are commonly reported in ampullary adenocarcinomas [51–53]. These findings may suggest a distinct molecular pathogenesis compared with other gastrointestinal NETs, possibly reflecting site-specific features related to the ampullary region.

Among potential actionable genomic alterations (AGAs), a fusion involving the *NTRK3* gene was identified in one of our cases. *NTRK3* fusions have been rarely described in NETs, occurring in approximately 0.1%–0.3% of cases [54, 55], with occasional reports in Pan-NETs [56] and pulmonary NETs [57]. Immunohistochemistry for pan-TRK performed in the fusion-positive case was negative; however, it has been reported that immunohistochemistry has lower sensitivity for *NTRK3* rearrangements compared to *NTRK1* and *NTRK2*.

A relevant observation of this study is the high frequency of AGAs involving kinase pathway-related genes (*HRAS, KRAS, NF1, CDK12,* and *NTRK3*), identified in 64% of SAMSOM-NETs. Examples include *HRAS* alterations, which may represent therapeutic targets for farnesyltransferase inhibitors (FTIs) (e.g., tipifarnib), and *NTRK* fusions, which are tumor-agnostic targets for *NTRK* inhibitors (e.g., larotrectinib and entrectinib).

Unlike Pan-NETs, which are frequently characterized by alterations in tumor suppressor genes such as *MEN1*, *DAXX*, and *ATRX* [12, 50], SAMSOM-NETs in our series lacked these molecular alterations and instead harbored recurrent *HRAS* mutations, suggesting a distinct molecular pathway of tumorigenesis. Moreover, this distinctive molecular profile may provide a valuable complementary tool for assigning the primary site in metastatic NETs of uncertain origin, particularly when integrated with clinical, morphological, and immunohistochemical features. However, molecular studies specifically investigating pancreatic somatostatinomas remain limited, precluding a direct comparison with this specific subset of Pan-NETs.

SAMSOM-NETs also appear to differ genomically from sporadic duodenal gastrinomas, in which *MEN1* mutations have been reported in approximately 40% of cases [58]. Furthermore, SAMSOM-NETs do not exhibit alterations in the WNT pathway (e.g., *CTNNB1* mutations or aberrant nuclear β-catenin expression), which have been reported in a subset of duodenal NETs [59, 60]. Nevertheless, the molecular landscape of the different histological subtypes of duodenal NETs remains poorly characterized and warrants dedicated future studies.

Several limitations of our study should be recognized. The sample size is limited; however, the present work represents an exploratory study based on a relatively large and rigorously selected series of an extremely rare neoplasm. Another limitation of our study is the lack of molecular profiling of metastatic lesions. Although all cases with liver metastases harbored RAS-pathway alterations in the primary tumor, metastatic tissue was not available for NGS analysis, precluding paired primary–metastasis genomic comparisons and the assessment of potential metastasis-specific molecular events. Therefore, the association between RAS-pathway alterations and metastatic behavior should be considered hypothesis-generating and requires validation in larger cohorts with available paired samples. In addition, a direct molecular comparison with a control group of duodeno-ampullary NETs lacking somatostatin expression was not feasible. Genome-wide molecular analysis, such as exome or genome sequencing, and gene expression profiling were not performed, nor was germline genetic testing carried out in all cases to exclude an underlying hereditary predisposition. *EPAS1* was not included in the gene panel used in the analysis of our cohort; therefore, the presence of potential alterations of this gene cannot be completely excluded. However, the examined patients did not exhibit clinicopathological features typical of Pacak–Zhuang syndrome (e.g., polycythemia or a history of paraganglioma/pheochromocytoma), and cases were negative for α-inhibin expression, a marker considered indicative for the pseudohypoxic pathway [61]. Finally, proteomic analysis of amyloid deposition was not performed.

In conclusion, our study indicates that SAMSOM-NETs represent a distinct subtype of NET characterized by peculiar histological and molecular features, including a characteristic paranuclear dot-like CK expression pattern, and recurrent alterations in the RAS pathway. These findings may have potentially relevant implications for the differential diagnosis with other tumor entities arising in this anatomical region and for the development of targeted therapeutic strategies.

## Supplementary Information

Below is the link to the electronic supplementary material.

Supplementary file1 (PNG 5390 KB)

Supplementary file2 (DOCX 220 KB)

## Data Availability

The data that support the findings of this study are not openly available due to reasons of sensitivity and are available from the corresponding author upon reasonable request.

## Declarations

### The use of AI-assisted Technologies

During manuscript preparation, the authors used ChatGPT (OpenAI Ireland Ltd., Dublin, Ireland) exclusively for linguistic support and spelling correction. All generated suggestions were carefully reviewed, revised where necessary, and fully verified by the authors, who retain complete responsibility for the final content of the manuscript.
