# Supplementary material for: Comprehensive Clinicopathologic, Immunohistochemical, and Genomic Profiling of Sporadic Ampullary Somatostatin-producing D-cell Neuroendocrine Tumors Identifies Recurrent HRAS Hotspot Mutations
Source: Endocr Pathol. 2026 Sep 12;37(1):38. doi: 10.1007/s12022-026-09934-y (PMC13570915; doi:10.1007/s12022-026-09934-y)
Supplement: Supplementary file 1 [file 12022_2026_9934_MOESM2_ESM.docx]

| **Antibody** | **Monoclonal/Polyclonal** | **Clone** | **Source** |
| --- | --- | --- | --- |
| Ki67 | Monoclonal | 30-9 | Roche |
| Chromogranin A | Monoclonal | LK2H10 | Roche |
| Synaptophysin | Monoclonal | SP11 | Roche |
| Insulinoma-associated protein 1 (INSM1) | Monoclonal | A-8 | Santa Cruz Biotechnology |
| Somatostatin Receptor type 2A (SSTR2A) | Monoclonal | UMB1 | Abcam |
| Cytokeratin CAM5.2 | Monoclonal | CAM5.2 | Roche |
| Pancytokeratin AE1/AE3 | Monoclonal | AE1/AE3/PCK26 | Roche |
| Cytokeratin 7 | Monoclonal | SP52 | Roche |
| Cytokeratin 20 | Monoclonal | SP33 | Roche |
| MUC1/Epithelial Membrane Antigen(EMA) | Monoclonal | E29 | Roche |
| Carcinoembryonic Antigen (CEA) | Monoclonal | II-7 | Dako |
| BCL10 | Monoclonal | 331.3 | Santa Cruz Biotechnology |
| MUC5AC | Monoclonal | MRQ-19 | Cell Marque |
| MUC6 | Monoclonal | MRQ-20 | Cell Marque |
| MUC2 | Monoclonal | MRQ-18 | Cell Marque |
| S100 Protein | Monoclonal | 4C4.9 | Roche |
| SOX10 | Monoclonal | SP267 | Cell Marque |
| Neurofilament protein | Monoclonal | 2F11 | Cell Marque |
| GATA3 | Monoclonal | L50-823 | Cell Marque |
| Tyrosine Hydroxylase(TH) | Monoclonal | EP1532Y | Abcam |
| PHOX2B | Monoclonal | EPR14423 | Abcam |
| ARX | Monoclonal | 11F6.2 | Merck |
| ISL1 | Monoclonal | EP283 | Cell Marque |
| CDX2 | Monoclonal | EPR2764Y | Cell Marque |
| SATB2 | Monoclonal | EP281 | Cell Marque |
| PDX1 | Polyclonal | — | Santa Cruz Biotechnology |
| Progesterone Receptor (PR) | Monoclonal | 1E2 | Roche |
| Estrogen Receptor (ER) | Monoclonal | SP1 | Roche |
| TTF-1 | Monoclonal | 8G7G3/1 | Roche |
| Gastrin | Polyclonal | — | Cell Marque |
| Somatostatin | Monoclonal | EP130 | Cell Marque |
| Serotonin | Monoclonal | EPR24431-201 | Abcam |
| Pancreatic polypeptide (PP) | Polyclonal | — | Diagnostic BioSystem |
| Glucagon | Monoclonal | EP74 | Cell Marque |
| Insulin | Monoclonal | BSB-42 | Bio SB |
| Calcitonin | Monoclonal | SP17 | Cell Marque |
| Succinate Dehydrogenase-B (SDHB) | Polyclonal | — | Atlas |
| ∝-inhibin | Monoclonal | MRQ-63 | Cell Marque |
| β-catenin | Monoclonal | 14 | Cell Marque |
| E-cadherin | Monoclonal | 36 | Dako |

**Supplementary Table 1.** Antibodies used for immunohistochemical analysis
